# Adherence to Surgical Antimicrobial Prophylaxis Guidelines and Improvement Efforts: A Single‐Center, Pre‐ and Post‐Intervention Comparison Study

**DOI:** 10.1002/hsr2.71516

**Published:** 2026-01-07

**Authors:** Chie Yamamoto, Yoko Nukui, Tadashi Kosaka, Kazuhiro Aoto, Rina Kozen, Kayo Hasegawa, Chikae Ota, Yuki Inoue, Masashi Taniguchi, Ryosuke Hamashima, Keitaro Furukawa, Keisuke Kikuchi, Ayami Nakanishi, Satoshi Teramukai, Yasuhiko Horii, Teiji Sawa

**Affiliations:** ^1^ Department of Infection Control and Laboratory Medicine Kyoto Prefectural University of Medicine Kyoto Japan; ^2^ Department of Pharmacy, University Hospital Kyoto Prefectural University of Medicine Kyoto Japan; ^3^ Central Surgical Unit/Local Anesthesia Surgery Center Kyoto Prefectural University of Medicine Kyoto Japan; ^4^ Department of Infectious Disease Kyoto City Hospital Kyoto Japan; ^5^ Department of Infection Control, University Hospital Kyoto Prefectural University of Medicine Kyoto Japan; ^6^ Department of Biostatistics, Graduate School of Medical Science Kyoto Prefectural University of Medicine Kyoto Japan; ^7^ Department of Anesthesiology Kyoto Prefectural University of Medicine Kyoto Japan

**Keywords:** antibacterial agents, antimicrobial stewardship, surgical antimicrobial prophylaxis, surgical site infections

## Abstract

**Background and Aims:**

Adherence to surgical antimicrobial prophylaxis (SAP) guidelines reduces the incidence of surgical site infections (SSIs) and improves prognosis and healthcare economics. This single‐center, pre‐ and post‐intervention comparative study investigated SAP guidelines adherence. Accordingly, we intervened where improvements were required. Changes in SAP guidelines adherence and SSI incidence were then evaluated.

**Methods:**

We assessed adherence to the appropriate antimicrobial administration timing within 1 h before skin incision in surgeries performed between April 2021 and June 2022. Regarding drug selection, dosage, and duration, we evaluated SAP protocols for 228 procedures across 18 departments and compared them with established guidelines.

**Results:**

Overall adherence to appropriate antimicrobial administration timing was 92.3% (4687/5076). Adherence was particularly low after gastroenterological surgery, cardiovascular surgery, orthopedic surgery, and neurosurgery, with a total adherence rate of 82.6% (1603/1941) across these four departments. Drug selection and duration deviated from guidelines in 25% (57/228) of procedures. The most common area requiring improvement was inappropriate drug selection in 57.9% (33/57), followed by unnecessarily long duration in 50.9% (29/57) of cases. In addition to an educational approach, a new antimicrobial initiation index, based on preoperative preparatory events, was established in low‐adherence departments. All protocols requiring improvement were revised to align with established guidelines. Consequently, adherence to the appropriate antimicrobial administration timing in departments with previously low adherence improved significantly (pre‐intervention: 82.6%; 95% confidence interval [CI]: 80.8%–84.2%; post‐intervention: 95.0%; 95% CI: 93.8%–96.0%; *p* < 0.001). Substantial reduction in SSI incidence was found in cardiovascular surgery (pre‐intervention: 6.9%; 95% CI: 3.2%–12.7%; post‐intervention: 0%; 95% CI: 0%–3.0%; *p* = 0.006).

**Conclusion:**

Establishing department‐specific antimicrobial initiation indexes, based on the surgical preparation stage, might be an effective measure for improving adherence to the appropriate antimicrobial administration timing in patients receiving SAP. Changes in SSI incidence require further study.

## Summary

- Adherence to surgical antimicrobial prophylaxis guidelines reduces the incidence of surgical site infections and improves patient prognosis and healthcare economics.
- In this single‐center, pre‐ and post‐intervention comparison study, adherence to surgical antimicrobial prophylaxis guidelines was investigated. Based on the results, a department‐specific antimicrobial initiation index was established for departments with low adherence to the appropriate timing of antimicrobial administration, leading to significant improvement. All protocols that deviated from the guidelines were revised through antimicrobial stewardship.
- The establishment of department‐specific antimicrobial initiation indices could be an effective measure for improving adherence to the appropriate timing of antimicrobial administration.

## 1 Introduction

Surgical site infections (SSIs) account for approximately 20% of all healthcare‐associated infections (HAIs) and are now among the most frequent [1, 2]. The incidence of SSIs for all surgical procedures in Japan is 3.8%; however, for some gastrointestinal tract surgeries, it can be as high as 22.9% [3]. Patients with an SSI have a 2–11 times higher risk of death compared with operative patients without SSIs. Additionally, 77% of deaths in patients with SSIs are directly attributable to the SSI [1]. In addition, SSIs are associated with prolonged hospital stays and increased readmission rates [1, 4]. In Japan, the occurrence of SSI prolongs hospital stays by 6.7–48.9 days [5]. Furthermore, SSIs generate $3.5 billion to $10 billion in excess medical costs annually, making them one of the costliest HAIs [1]. However, 31%–84% of SSIs are preventable and require adherence to crucial preventive measures, including surgical antimicrobial prophylaxis (SAP) [1, 6]. In patients receiving SAP, appropriate antimicrobials must be administered within 1 h before skin incision to reach bactericidal blood or tissue concentrations at the time of incision. Deviations from the appropriate timing of antimicrobial administration can increase SSI incidence [7, 8, 9]. Furthermore, unnecessary prolongation of SAP after surgery and the use of broad‐spectrum antimicrobial agents do not reduce SSI incidence and are associated with potential adverse drug reactions, antimicrobial resistance, superinfections, and additional costs [7, 10]. The objective of this study was to investigate adherence to SAP guidelines at our institution and intervene in collaboration with the Antimicrobial Stewardship Team (AST) and each clinical department/division where improvements were required, and evaluate the impact of these interventions.

## 2 Material and Methods

### 2.1 Study Design and Setting

This single‐center, pre‐ and post‐intervention comparison study was conducted at the Kyoto Prefectural University of Medicine Hospital, an acute‐care hospital in Kyoto City (population approximately 1.44 million). This study adhered to the SQUIRE 2.0 (Standards for Quality Improvement Reporting Excellence): revised publication guidelines from a detailed consensus process for reporting quality improvement studies. Ethical approval was obtained from the Institutional Review Board of the Kyoto Prefectural University of Medicine (approval number: ERB‐C‐3134, April 9, 2024). Informed consent was obtained in the form of opt‐out on the website (https://www.kpum-rinken.com/kansenkensa). Those who declined were excluded. The study was led by an AST comprising multidisciplinary professionals, including infectious disease physicians, pharmacists, and infection control nurses. Furthermore, Kyoto Prefectural University of Medicine Hospital has designated infection control liaisons in all departments and divisions, distinct from the head of the department, primarily focusing on staff with 10 or more years of experience. These liaisons routinely collaborate with the AST and Infection Control Team to prevent HAIs. Their collective cooperation facilitated the current study. We first assessed adherence to SAP guidelines, including the timing of antimicrobial administration within 1 h before skin incision, drug selection, dosage, and duration. According to the timing of antimicrobial administration, the perioperative patient information system: ORSYS (Philips Electronics Japan, Tokyo, Japan) was used as a reference. Regarding drug selection, dosage, and duration, we evaluated SAP protocols within each surgical procedure and department and compared them with the established guidelines [11, 12, 13]. The practices recommended by these guidelines were defined as appropriate, whereas all other practices were considered inappropriate. Next, based on the adherence to SAP guidelines, we intervened in areas where improvements were required and evaluated the effects of these interventions. The primary endpoints for evaluating the effectiveness of the interventions were changes in adherence to SAP guidelines, particularly the appropriate timing of antimicrobial administration within 1 h before skin incision. The secondary endpoints were SSI incidence before and after intervention. SSIs included superficial incisional wound infections, deep incisional wound infections, and organ and body cavity infections. They were defined as occurring within 30 days postoperatively for non‐prosthetic procedures and within 1 year postoperatively for prosthetic procedures [13]. The SSI incidence rate was calculated for each surgical procedure as (number of SSI/total number of surgeries) × 100 during the study period in accordance with the criteria of the Japan Nosocomial Infections Surveillance [3].

### 2.2 Inclusion and Exclusion Criteria

Regarding the timing of antimicrobial administration, we evaluated surgeries performed under general anesthesia during the study period that were eligible for inclusion, without distinction based on urgency (elective vs. emergency) or wound classification (clean, clean‐contaminated, or contaminated/dirty). Surgeries performed under local anesthesia were excluded. Surgeries that required SAP with vancomycin and quinolones, which must be administered 2 h before skin incision, were also excluded. Regarding drug selection, dosage, and duration, we evaluated the SAP protocols for 228 procedures across 18 departments, which were actively utilized as of April 2022. Surgical procedures to which no response was obtained from the respective departments or for which a specific protocol was not established were excluded. Cardiovascular surgery (open‐heart surgery and coronary artery bypass grafting) and neurosurgery (craniotomy), which were originally under surveillance, were included in the estimation of SSI incidence.

### 2.3 Intervention Details

Appropriate timing of antimicrobial administration: The AST provided a lecture to anesthesiologists and operating room nurses, specifically targeting their infection control liaisons, emphasizing the importance of adherence to appropriate timing of antimicrobial administration within 1 h before skin incision. This information was subsequently disseminated within their respective departments and divisions by these liaisons. Furthermore, the preoperative checklist for each patient, which must be confirmed by the attending physician, ward nurses, operating room nurses, and anesthesiologists, was revised. Specifically, the entry for “pre‐skin incision antimicrobial” was changed to “antimicrobial administered within one hour prior to skin incision” to raise awareness about appropriate administration timing. Additional interventions were targeted at departments with low adherence. During the pre‐intervention period, antimicrobials were administered at the time of anesthesia induction in almost all procedures. Regarding departments with low adherence, we referred to the perioperative patient information system to identify events within the surgical preparation phase that occurred within 1 h before skin incision. These identified events, rather than anesthesia induction, were then defined as the department‐specific antimicrobial initiation indices. These new indices were made known to all operating room nurses and anesthesiologists and reconfirmed at daily preoperative meetings. A double‐check was conducted when operating room nurses handed antimicrobials to the anesthesiologists.

Drug selection, dosage, and duration: SAP protocols, specific to each surgical procedure within individual departments, were submitted to the AST. The AST members meticulously checked each protocol for adherence to established guidelines for drug selection, dosage, and duration. Considering protocols with identified deviations, the AST first held internal discussions to develop revisions aligned with the guidelines. Subsequently, AST members presented these revised protocols to the respective department's infection control liaisons. During these presentations, it was explained that unnecessarily broad‐spectrum and prolonged antimicrobials did not contribute to reducing SSI and could instead lead to adverse events, drug resistance, superinfections, and excessive costs. The content of these presentations has since been disseminated to all physicians in each department via their respective infection control liaisons. Following extensive discussions within each department, among AST members, and with the infection control liaisons of each department, revised protocols were developed.

These investigations for adherence to SAP guidelines and interventions were implemented over a 3‐month period from April to June 2022; the new antimicrobial initiation indices and revised SAP protocols were subsequently launched in July 2022. We defined April 2021 to June 2022 as the pre‐intervention period and July 2022 to June 2023 as the post‐intervention period.

Data are presented as numbers and percentages, as appropriate, and the results are presented in text, tables, and figures. Categorical variables were compared using Fisher's exact test to determine the association among them. Two‐sided tests were used for the analysis, and statistical significance was set at *p* < 0.05. Statistical analyses were performed using EZR version 1.63 (Saitama Medical Center, Jichi Medical University, Saitama, Japan).

## 3 Results

### 3.1 Adherence to SAP Guidelines Pre‐Intervention

Overall, 5076 surgeries were performed during the pre‐intervention period. In 4687 surgeries (92.3%), antimicrobial administration was appropriate. However, in 369 surgeries (7.3%), antimicrobials were administered at inappropriate times, either more than 1 h before or after skin incision. In 20 surgeries (0.4%), the timing of antimicrobial administration was unknown due to omissions or other reasons. Adherence was particularly low in gastroenterological surgery, cardiovascular surgery, orthopedic surgery, and neurosurgery, with a total adherence rate of 82.6% (1603/1941) across these four departments. In four departments, the preparation time before surgery was longer, with skin incisions occurring more than 1 h from anesthesia induction. We surveyed events before skin incision based on perioperative patient information systems and observed that positional immobilization in gastrointestinal surgery, central venous catheter insertion in cardiovascular surgery, physician handwashing in orthopedic surgery, and skin incision marking in neurosurgery were all performed within 1 h before skin incision. Therefore, these events were defined as new antimicrobial initiation indices. Drug selection and duration deviated from guidelines in 25% (57/228) of procedures. Among these, 33 procedures (57.9%) required a revision in drug selection. Among inappropriate drug selections, fluoroquinolones were the most frequent choice, accounting for 30.3% (10/33), followed by second‐generation cephalosporins at 24.2% (8/33) and β‐lactamase inhibitor‐combined penicillin at 18.2% (6/33). Overall, 72.7% (24/33) involved the selection of broad‐spectrum antimicrobials other than those recommended. Furthermore, by surgical category, clean‐contaminated surgeries accounted for 45.5% (15/33), followed by clean surgeries at 42.4% (14/33) and contaminated/dirty surgeries at 12.1% (4/33). Moreover, 29 (50.9%) required a shorter duration of SAP. In thoracic surgery and interventional radiology, all registered procedures exhibited inappropriate duration, with administration continuing for 24–48 h postoperatively despite being indicated for a single dose. Furthermore, plastic surgery and urology exhibited a longer duration of antimicrobial administration, with 50% (3/6) and 36.4% (8/22) of their registered procedures, respectively, receiving postoperative antimicrobial therapy for 3–8 days despite a single dose being originally recommended. By surgical categories, clean surgeries constituted the largest proportion at 51.7% (15/29), followed by clean‐contaminated surgeries at 41.4% (12/29) and contaminated/dirty surgeries at 6.9% (2/29). Additionally, a new alternative was needed for 16 procedures (28.1%) due to beta‐lactam allergy. Multiple revisions, involving a combination of two or more areas, were required in 22 procedures (38.6%). No deviations from the guidelines were observed for the dosage of antimicrobials.

### 3.2 Post‐Intervention Results

**Figure 1:**
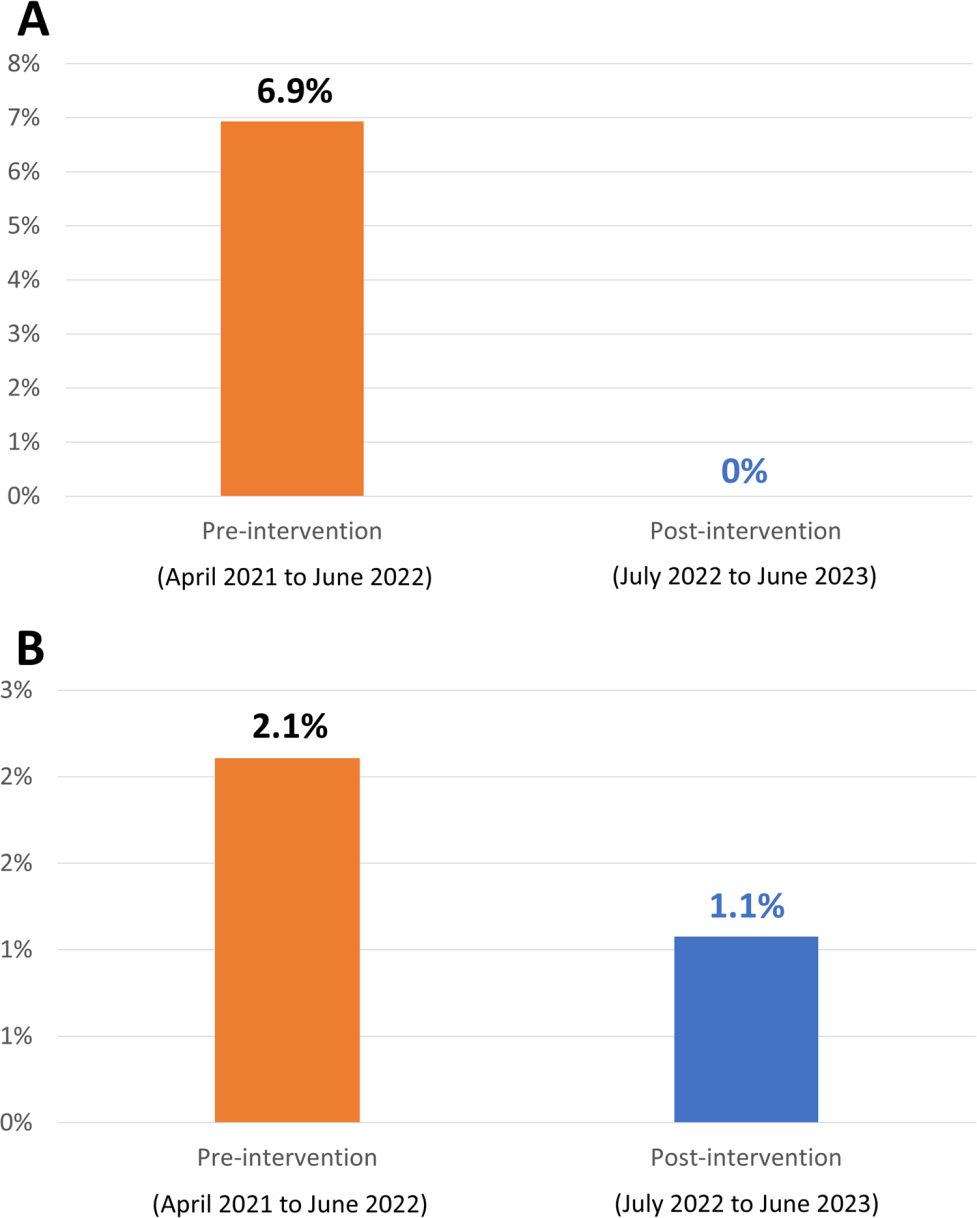
Incidence of surgical site infections after (A) cardiovascular surgery (open‐heart surgery and coronary artery bypass grafting) and (B) neurosurgery (craniotomy) pre‐ and post‐intervention.

We then compared the adherence to the appropriate timing of antimicrobial administration in the four departments with low adherence, adherence to guidelines in SAP protocols among drug selection, and duration and incidence of SSIs before and after the intervention. Consequently, adherence to the appropriate timing of antimicrobial administration in these four departments significantly improved (pre‐intervention: 82.6%; 95% confidence interval [CI]: 80.8%–84.2%; post‐intervention: 95.0%; 95% CI: 93.8%–96.0%; *p* < 0.001) (Table 1). All SAP protocols of 57 procedures that deviated from the guidelines were revised to align with the guidelines. The surgical clinical path was revised in most cases. SSI incidence was significantly reduced in cardiovascular surgery (pre‐intervention: 6.9%; 95% CI: 3.2%–12.7%; post‐intervention: 0%; 95% CI: 0%–2.9%; *p* = 0.006). SSI incidence was halved in neurosurgery (pre‐intervention: 2.1%; 95% CI: 0.26%–7.4%; post‐intervention: 1.1%; 95% CI: 0.03%–5.8%; *p* > 0.99) (Figure 1, Table S1).

**Table 1:** Number of pre‐intervention surgeries and adherence to the appropriate timing for administering surgical antimicrobial prophylaxis by department.

Department	Number of surgeries and timing of antimicrobial administration	Pre‐intervention	Post‐intervention	*p*‐value
Gastroenterological surgery	Number of surgeries in which antimicrobials were administrated at the appropriate timing	561	477	< 0.001
	Total number of surgeries	602	487	
	Adherence rate to appropriate timing of antimicrobial administration	93.2% (90.5%–95.1%)	97.9% (96.3%–99.0%)	
Cardiovascular surgery	Number of surgeries in which antimicrobials were administrated at the appropriate timing	239	235	< 0.001
	Total number of surgeries	316	249	
	Adherence rate to appropriate timing of antimicrobial administration	75.6% (70.5%–80.3%)	94.4% (90.7%–96.9%)	
Orthopedic surgery	Number of surgeries in which antimicrobials were administrated at the appropriate timing	723	663	< 0.001
	Total number of surgeries	855	700	
	Adherence rate to appropriate timing of antimicrobial administration	84.6% (82.0%–86.9%)	94.7% (92.8%–96.3%)	
Neurosurgery	Number of surgeries in which antimicrobials were administrated at the appropriate timing	80	143	< 0.001
	Total number of surgeries	168	162	
	Adherence rate to appropriate timing of antimicrobial administration	47.6% (40.0%–55.5%)	88.3% (83.3%–92.2%)	
Total	Number of surgeries in which antimicrobials were administrated at the appropriate timing	1603	1518	< 0.001
	Total number of surgeries	1941	1598	
	Adherence rate to appropriate timing of antimicrobial administration	82.6% (80.8%–84.2%)	95.0% (93.8%–96.0%)	

*Note:* Data are presented as *n*; Values in parentheses represent the 95% confidence interval (CI).

## 4 Discussion

Non‐adherence to SAP guidelines was associated with an increased SSI incidence, regardless of the procedure [14]. However, adherence to SAP guidelines was not uniform, ranging from 3.0% to 64.0% for the appropriate timing of antimicrobial administration, 12.7% to 100% for drug selection, and 22% to 95% for indicated discontinuation, with rates of discontinuation varying from 4.6% to 91.4% [15, 16, 17]. In factors contributing to non‐adherence, associations have been noted with underlying disease, length of preoperative hospitalization, extended surgical duration, institution, and department of surgery [10, 17]. Interventions based on antimicrobial stewardship improve adherence to guidelines, thereby reducing SSI incidence, hospital stay length, readmission rates, and improving healthcare economics. The current study investigated adherence to SAP guidelines. Moreover, we intervened where improvements were required. First, we determined the new antimicrobial initiation index for each department with low adherence based on preoperative events. Furthermore, the new index was shared and regularly checked by each professional involved in surgeries to facilitate adherence. In previous studies evaluating adherence to the timing of antimicrobial administration, improvement measures included education, audits, and feedback; revision of protocols; and the application of computer‐assisted decisions. Of these, specific improvement measures included distributing pocket cards with the clinical path to staff, posting protocols in the operating room, and automating computerized antimicrobial administration times [14, 18, 19, 20, 21]. No reports exist on the establishment of department‐specific antimicrobial administration indices based on preoperative preparatory events, as in the current study. The importance of education, caution, audits, and feedback cannot be overstated. However, there remains a concern that these interventions may not be communicated to all staff. Although revising protocols and automating antimicrobial administration timing using computer‐assisted systems is effective, each department has different preparations; unforeseen events often lead to changes in the surgery start time. We have significantly improved adherence to the appropriate timing of antimicrobial administration by implementing more structured interventions. Drug selection and duration were revised to comply with the guidelines for all procedures that required improvement. Lower adherence rates were observed in terms of drug selection (57.9%) and SAP duration (50.9%), similar to the findings in a previous study [10, 17].

This study has certain limitations. Since this was a single‐center study, adherence to SAP guidelines and SSI incidence were evaluated in a limited setting with specific procedures. Furthermore, Fiallo et al. reported that 41.3% of surgical cases had already been administered antimicrobials for the treatment of infections in the target organs, and SAP was administered excessively [22]. The current study did not evaluate the overlap between antimicrobials for therapeutic purposes and SAP. Addressing these issues constitutes a crucial challenge, necessitating further investigation. Furthermore, we could not assess the involvement of other factors, such as patient characteristics, surgical procedure or duration, and surgical wound management, which might have contributed to SSI incidence. Therefore, further studies are warranted.

## 5 Conclusion

We presented adherence to SAP guidelines and improvement efforts. The study suggests that, in addition to an educational approach, the establishment of department‐specific antimicrobial initiation indexes based on the stage of surgical preparation may be an effective measure for improving adherence to the appropriate timing of antimicrobial administration in patients receiving SAP.

## Author Contributions

**Chie Yamamoto:** writing – original draft, methodology, validation, formal analysis, investigation, visualization, writing – review and editing, project administration. **Yoko Nukui:** conceptualization, methodology, validation, formal analysis, investigation, writing – review and editing, project administration. **Tadashi Kosaka:** software, resources, data curation. **Kazuhiro Aoto:** methodology. **Rina Kozen:** methodology. **Kayo Hasegawa:** project administration. **Chikae Ota:** project administration. **Yuki Inoue:** project administration. **Masashi Taniguchi:** validation, project administration. **Ryosuke Hamashima:** project administration. **Keitaro Furukawa:** project administration. **Keisuke Kikuchi:** validation, project administration. **Ayami Nakanishi:** validation, project administration. **Satoshi Teramukai:** formal analysis, supervision. **Yasuhiko Horii:** resources, supervision, project administration. **Teiji Sawa:** resources, supervision.

## Ethics Statement

This study was approved by the Institutional Review Board of the Kyoto Prefectural University of Medicine (approval number: ERB‐C‐3134, April 9, 2024).

## Consent

Informed consent was obtained in the form of opt‐out on the website. Those who declined were excluded.

## Conflicts of Interest

The authors declare no conflicts of interest.

## Transparency Statement

The lead author, Chie Yamamoto, affirms that this manuscript is an honest, accurate, and transparent account of the study being reported; that no important aspects of the study have been omitted; and that any discrepancies from the study as planned (and, if relevant, registered) have been explained.

## Supporting information

Supplementary Table 1 0708.

## Data Availability

The data that support the findings of this study are available in the supporting material of this article.

## References

[1] M. S. Calderwood , D. J. Anderson , D. W. Bratzler , et al., “Strategies to Prevent Surgical Site Infections in Acute‐Care Hospitals: 2022 Update,” Infection Control & Hospital Epidemiology 44 (2023): 695–720, 10.1017/ice.2023.67. 37137483 PMC10867741

[2] A. Versporten , P. Zarb , I. Caniaux , et al., “Antimicrobial Consumption and Resistance in Adult Hospital Inpatients in 53 Countries: Results of an Internet‐Based Global Point Prevalence Survey,” Lancet Global Health 6 (2018): e619–e629, Erratum in: *Lancet Global Health* 6, no. 9 (2018):e968, 10.1016/S2214-109X(18)30186-4. 29681513

[3] JANIS, *Japan Nosocomial Infections Surveillance* (2024, Ministry of Health, Labour and Welfare), accessed June 21, 2025, https://janis.mhlw.go.jp/report/ssi.html.

[4] S. Shambhu , A. S. Gordon , Y. Liu , et al., “The Burden of Health Care Utilization, Cost, and Mortality Associated With Select Surgical Site Infections,” Joint Commission Journal on Quality and Patient Safety 50 (2024): 857–866, 10.1016/j.jcjq.2024.08.005. 39384467

[5] K. Morikane , “Epidemiology and Prevention of Surgical Site Infection in Japan,” Journal of Hospital Infection 146 (2024): 192–198, 10.1016/j.jhin.2023.10.027. 38369060

[6] P. W. Schreiber , H. Sax , A. Wolfensberger , L. Clack , and S. P. Kuster , “The Preventable Proportion of Healthcare‐Associated Infections 2005–2016: Systematic Review and Meta‐Analysis,” Infection Control & Hospital Epidemiology 39 (2018): 1277–1295, 10.1017/ice.2018.183. 30234463

[7] M. E. E. van Kasteren , J. Mannien , A. Ott , B. J. Kullberg , A. S. de Boer , and I. C. Gyssens , “Antibiotic Prophylaxis and the Risk of Surgical Site Infections Following Total Hip Arthroplasty: Timely Administration Is the Most Important Factor,” Clinical Infectious Diseases 44 (2007): 921–927, 10.1086/512192. 17342642

[8] R. Sommerstein , N. Troillet , S. Harbarth , et al., “Timing of Cefuroxime Surgical Antimicrobial Prophylaxis and Its Association With Surgical Site Infections,” JAMA Network Open 6 (2023): e2317370, 10.1001/jamanetworkopen.2023.17370. 37289455 PMC10251212

[9] S. W. de Jonge , S. L. Gans , J. J. Atema , J. S. Solomkin , P. E. Dellinger , and M. A. Boermeester , “Timing of Preoperative Antibiotic Prophylaxis in 54,552 Patients and the Risk of Surgical Site Infection: A Systematic Review and Meta‐Analysis,” Medicine 96 (2017): e6903, 10.1097/MD.0000000000006903. 28723736 PMC5521876

[10] L. B. Pereira , C. S. Feliciano , F. Bellissimo‐Rodrigues , and L. R. L. Pereira , “Evaluation of the Adherence to Surgical Antibiotic Prophylaxis Recommendations and Associated Factors in a University Hospital: A Cross‐Sectional Study,” American Journal of Infection Control 52 (2024): 1320–1328, 10.1016/j.ajic.2024.07.004. 38996873

[11] Advisory Committee of Clinical Practice Guidelines for Antimicrobial Prophylaxis in Surgery, “ Japanese Clinical Practice Guidelines for Antimicrobial Prophylaxis in Surgery,” Journal of Japan Society for Surgical Infection 17 (2020): 154–165, 10.1089/sur.2013.9999.

[12] World Health Organization, *Global Guidelines for the Prevention of Surgical Site Infection* (2nd ed.) (2018, World Health Organization), accessed June 21, 2025, https://www.who.int/publications/i/item/9789241550475.

[13] S. I. Berríos‐Torres , C. A. Umscheid , D. W. Bratzler , et al., “Centers for Disease Control and Prevention Guideline for the Prevention of Surgical Site Infection, 2017,” JAMA Surgery 152 (2017): 784–791, Erratum in: *JAMA Surgery* 152, no. 8 (2017): 803, 10.1001/jamasurg.2017.0904. 28467526

[14] J. V. Martinez‐Sobalvarro , A. A. P. Júnior , L. B. Pereira , A. O. Baldoni , C. S. Ceron , and T. M. dos Reis , “Antimicrobial Stewardship for Surgical Antibiotic Prophylaxis and Surgical Site Infections: A Systematic Review,” International Journal of Clinical Pharmacy 44 (2022): 301–319, 10.1007/s11096-021-01358-4. 34843035

[15] M. Gouvêa , C. O. Novaes , D. M. T. Pereira , and A. C. Iglesias , “Adherence to Guidelines for Surgical Antibiotic Prophylaxis: A Review,” Brazilian Journal of Infectious Diseases 19 (2015): 517–524, 10.1016/j.bjid.2015.06.004. PMC9427538 26254691

[16] H. Morioka , Y. Koizumi , T. Watariguchi , et al., “Surgical Antimicrobial Prophylaxis in Japanese Hospitals: Real Status and Challenges,” Journal of Infection and Chemotherapy 30 (2024): 626–632, 10.1016/j.jiac.2024.01.013. 38272262

[17] H. Morioka , H. Ohge , M. Nagao , et al., “Appropriateness of Surgical Antimicrobial Prophylaxis in Japanese University Hospitals,” Journal of Hospital Infection 129 (2022): 189–197, 10.1016/j.jhin.2022.06.017. 35835283

[18] L. Naseralallah , S. Koraysh , B. Aboujabal , and M. Alasmar , “Effectiveness of Pharmacist‐Led Antimicrobial Stewardship Programs in Perioperative Settings: A Systematic Review and Meta‐Analysis,” Research in Social and Administrative Pharmacy 20 (2024): 1023–1037, 10.1016/j.sapharm.2024.08.006. 39153871

[19] D. Donà , D. Luise , E. Barbieri , et al., “Effectiveness and Sustainability of an Antimicrobial Stewardship Program for Perioperative Prophylaxis in Pediatric Surgery,” Pathogens 9 (2020): 490, 10.3390/pathogens9060490. 32575542 PMC7350339

[20] T. Saied , S. F. Hafez , A. Kandeel , et al., “Antimicrobial Stewardship to Optimize the Use of Antimicrobials for Surgical Prophylaxis in Egypt: A Multicenter Pilot Intervention Study,” American Journal of Infection Control 43 (2015): e67–e71, 10.1016/j.ajic.2015.07.004. 26315059

[21] A. Ribed , B. Monje , X. García‐González , et al., “Improving Surgical Antibiotic Prophylaxis Adherence and Reducing Hospital Readmissions: A Bundle of Interventions Including Health Information Technologies,” European Journal of Hospital Pharmacy 27 (2020): 237–242, 10.1136/ejhpharm-2018-001666. 32587084 PMC7335616

[22] P. Fiallo , T. Williams , and L. M. Bush , “When Antimicrobial Treatment and Surgical Prophylaxis Collide: A Stewardship Opportunity,” Hospital Pharmacy 59 (2024): 460–464, 10.1177/00185787241230079. 38919764 PMC11195835

